# mHealth Interventions To Support Self-Management In HIV: A Systematic Review

**DOI:** 10.2174/1874613601711010119

**Published:** 2017-11-21

**Authors:** Vanessa Cooper, Jane Clatworthy, Jennifer Whetham, EmERGE Consortium

**Affiliations:** The Lawson Unit, Brighton and Sussex University Hospitals NHS Trust, Brighton, England

**Keywords:** mHealth, HIV treatment, Information technology, Mobile phone, Self-management

## Abstract

**Background:**

Self-management is an important aspect of long-term HIV treatment. Mobile technologies offer the potential to efficiently deliver interventions to facilitate HIV self-management. The last comprehensive review of such mHealth interventions was conducted in 2011. Given the rapidly evolving field, a need was identified for an updated review of the literature.

**Objective:**

The study aimed to describe and evaluate current evidence-based mHealth interventions to support self-management in HIV.

**Method:**

Eight online databases (Medline, Scopus, Embase, PsycINFO, Cochrane, Global Health CAB, IEEE explore, Web of Science) were systematically searched for papers describing and evaluating mHealth HIV self-management interventions. Reference lists of relevant papers were also searched. Data on intervention content and evaluation methodology were extracted and appraised by two researchers.

**Results:**

41 papers were identified evaluating 28 interventions. The majority of these interventions (n=20, 71%) had a single focus of either improving adherence (n=16), increasing engagement in care (n=3) or supporting smoking cessation (n=1), while just 8 (29%) were more complex self-management interventions, targeting a range of health-related behaviours. Interventions were predominantly delivered through SMS messaging. They significantly impacted on a range of outcomes including adherence, viral load, mental health and social support.

**Conclusion:**

Since the last major review of mHealth interventions in HIV, there has been a shift from exploratory acceptability/feasibility studies to impact evaluations. While overall the interventions impacted on a range of outcomes, they were generally limited in scope, failing to encompass many functions identified as desirable by people living with HIV. Participant incentives may limit the generalizability of findings.

## 1 INTRODUCTION

Antiretroviral therapy (ART) has transformed HIV from a disease with high mortality to a long-term condition. Life expectancy is now near normal in developed countries, provided that treatment is initiated early in the course of the illness and maintained over the long-term [1-3]. As people with HIV reach older age, they are vulnerable to multiple age-related comorbidities [4]. Given the need for long-term clinical monitoring of laboratory markers of disease (CD4 count and viral load), risk factors for age-related conditions, and adherence to medication, there is increasing focus on self-management among individuals living with HIV.

Evidence-based self-management interventions for people living with HIV (PLWH) have improved knowledge, physical health and both psychosocial and behavioural outcomes [5]. However face-to-face interventions are time and resource intensive, and may be difficult for most people to access. eHealth (strategies, tools and services using information and communication technologies) [6] have increased the accessibility of self-management interventions.

The use of mobile and wireless technologies to support the achievement of health objectives (mHealth) is the quickest growing area of eHealth, due to rapid advances in mobile applications and technologies and increasing coverage of mobile cellular networks [7]. In 2015, over half of the world’s population had a mobile phone subscription [8] and it has been estimated that by 2018 the majority of all mobile phone users will have smartphones [9]. mHealth has the potential to provide consistency in the delivery of interventions across a wide population at low cost [10, 11].

Mobile devices are capable of receiving reminders and educational messages, giving round the clock, real time feedback as well as opportunities to communicate with healthcare providers. Smartphones and tablet computers are also able to host applications (apps) with a multitude of capabilities such as social networking, gaming, and diagnostics [10, 12].

A design science perspective has been advocated when developing mHealth interventions to ensure that the product is safe, useful and effective [13]. This involves three iterative cycles; a relevance cycle to understand the requirements of the user, a rigor cycle drawing on and furthering the knowledge base and a design cycle developing and evaluating the intervention. As part of the relevance cycle, Schnall and colleagues formed focus groups with people living with HIV to identify key requirements of mHealth interventions. Participants highlighted the need for interventions that were useful, easy to use, had low risk (i.e. data stored securely, no risk of unwanted disclosure of HIV status), with trusted creators of the technology [14].They identified nine functions that would be encompassed within their ideal app: patient-provider and peer communication; medication and appointment reminders; a medication checklist including a pill identification function and a list of current and discontinued medicines; lab reports (CD4 count, viral load, sexually transmitted infections, glucose and complete blood count); pharmacy information; nutrition and fitness trackers; resources (such as links to social services, substance abuse support, video testimonials and case management); settings (profile picture, password and alerts); and a search function. The authors then reviewed apps that were currently available for PLWH. None of the apps included all of the desired functions, three-quarters had less than four functions and none included functions on nutrition or fitness [15].

In order to determine their validity and effectiveness, it is important that mHealth interventions are rigorously evaluated [16]. The findings of recent reviews [10, 11, 15, 17-21] reveal several limitations of studies evaluating mHealth interventions for HIV treatment and care. While there is evidence that weekly short message system (SMS) reminders can be effective for improving medication adherence [20], few studies have evaluated mHealth interventions that encompass other aspects of self-management, such as attendance of medical appointments, self-monitoring, information on demand, educational messaging, health promotion or diagnostics [10, 15, 17, 19]. The literature has been dominated by small-scale feasibility and exploratory studies and pilot evaluations that lack statistical power [10, 11, 17, 19], and the cost-effectiveness of mHealth interventions has not yet been ascertained [19, 21]. Moreover, many studies fail to articulate a theoretical framework and it is not possible to determine which aspects of the intervention were effective, or why [17]. Several reviews have highlighted a lack of research to establish the effectiveness of mHealth interventions focused on specific communities such as men who have sex with men, migrants, people who use injection drugs, women or adolescents [17, 19-21].

The most comprehensive review of HIV mHealth interventions to date covered articles published up to December 2011 [17]. Given that mHealth is a rapidly evolving field [16], we sought to identify research published since January 2012, in order to describe and evaluate current evidence-based mHealth interventions to support self-management in HIV. We had three specific research questions: 1) What types of intervention have been developed to support self-management among people living with HIV? 2) How effective are these interventions at improving self-management? 3) How feasible and acceptable are these interventions?

## 2 METHOD

Eight online databases (Pubmed/Medline, Scopus, Embase, PsycINFO, Cochrane, Global Health CAB, IEEE explore and Web of Science) were searched for relevant papers in October 2015. Search terms were developed based on those used by Catalani *et al.* [17]: (mHealth OR mobile phone* OR handheld device* OR cellular phone* OR mobile device* OR handheld computer* OR iPAD* OR android tablet* OR smart device* or smart phone*) AND (HIV or Human Immunodeficiency Virus). Searches were limited to January 2012 onwards as the review conducted by Catalani *et al.* covered the period up to December 2011. ClinicalTrials.gov was also searched using the same search terms to identify relevant studies in progress. These studies were followed up in October 2016 to see whether they had subsequently been published.

In addition to the electronic database search, reference lists of reviewed papers were searched for additional relevant material. References of retrieved articles were exported to EndNote and duplicates removed. The references and abstracts were then exported into an Excel spreadsheet for abstract review. This process was conducted by two researchers with 100% overlap. Disagreement was resolved through discussion. Full text copies of papers that appeared relevant were obtained and subjected to further scrutiny. Papers were included in the review if they met the following criteria:

Inclusion criteria

1: The article reports on the systematic investigation of a specific mHealth intervention
2: The mHealth intervention is HIV-focused
3: The article specifies the study design and provides a description of the methods used for investigation
4: The study assesses feasibility, acceptability, patient-related outcomes, adherence and/or cost-effectiveness

Exclusion criteria

1: mHealth is focused on the prevention of HIV
2: mHealth is focused on HIV screening
3: mHealth intervention was developed specifically for children
4: mHealth intervention was developed for health workers

Two researchers independently extracted the following data from the articles: country, study aim, method (quantitative or qualitative), design (cross-sectional, pre-post, RCT), population, sample size, description of the intervention (*e.g.* mode of delivery, functions, target behaviours), outcomes measures and results.

Two researchers (VC and JC) independently rated the quality of the retrieved articles, using the Mixed Methods Assessment Tool [MMAT] [22]. This tool enables the assessment of studies with a wide range of designs, including mixed methods studies. For each type of study (qualitative, RCT and quantitative descriptive studies), four items were used to assess quality. For mixed methods studies, the quality of both the qualitative and quantitative elements were assessed, as well as completing an additional three questions focusing on the integration of the methods. For each item, response categories were ‘yes’, ‘no’ or ‘can’t tell’. Each study received a score ranging from 25% (*) (1 criterion met) to 100% (****) (all criteria met). For mixed method studies, the overall quality score was derived from the lowest scoring of the qualitative, quantitative and mixed methods components. Disagreement between researchers was resolved through discussion. No study was excluded on the basis of the quality assessment because we were interested in collating information on all relevant mHealth interventions. The quality of published conference abstracts and poster presentations was not assessed due to insufficient information being provided to conduct the assessment.

## 3 RESULTS

### 3.1 Selection of Articles for the Review

The electronic database search identified 570 potentially relevant papers, 132 of which were selected for full text screening on the basis of the title and abstract search. These papers were obtained and subjected to full text review, with 37 papers meeting the inclusion criteria. Further 4 papers were identified from the reference list search and communication with authors, resulting in a total of 41 papers (see Table S1 for an overview). Reasons for exclusion at each stage are shown in Fig. (**1**). Four published protocols were identified using the electronic database search (n=3) and the search of ClinicalTrials.gov (n=1) (Table S2). Five further relevant ongoing studies were identified through ClinicalTrials.gov (Table S3). The results of these studies had not been published when followed up in October 2016.

### 3.2 Characteristics and Quality of Included Studies

The methodological characteristics of the 41 studies are summarized in Table S4. The majority of the studies used quantitative or mixed quantitative and qualitative methods. The studies were conducted in 12 countries across North America, South America, Africa, Asia, Europe and New Zealand. The majority of studies were conducted in North America (23; 56%). Two thirds of the articles (27; 66%) described studies conducted with specific groups of PLWH: (*e.g.* men who have sex with men; women; young people; people with or at risk of low adherence; smokers, heavy drinkers or drug users).

It was possible to assess the quality of 32 studies (the remaining 9 were conference abstracts or posters) using the MMAT, with scores ranging from 25% (*) to 100% (****) (see Table S1 for individual study scores). Overall, 20 (62%) of these studies scored 75% or 100%, indicating good quality. The quality of the studies including a qualitative component was generally poorer than that of the quantitative studies, with only 1 (20%) of 5 qualitative/mixed methods studies assessed scoring over 50%. None of the qualitative studies demonstrated appropriate consideration of how the findings might relate to the researcher’s influence (reflexivity).

#### 3.3 What types of intervention have been developed to support self-management among people living with HIV?

The 41 papers included in the review described 28 interventions. Key features of these interventions are presented in Table S5. The interventions were delivered via SMS text messaging (16; 57%), mobile applications (5; 18%), mobile telephone calls (3;11%), Interactive Voice Response (IVR – 1; 4%), and downloaded videos (1; 4%). In addition, one intervention combined SMS messaging with IVR and one offered a choice of delivery methods. Less than a third of the interventions were designed with an aim of impacting on multiple self-management behaviours (8; 29%). Most interventions instead focused on a single target behaviour, either adherence to medication (16; 57%), engagement with care (*e.g.* appointment attendance) (3; 11%) or smoking cessation (1; 4%). The interventions included a wide range of functions (n=15) to achieve these aims, the most common of which were medication reminders (16; 57%); behaviour monitoring (13; 46%); patient-provider communication (10; 36%); personalized feedback (8; 29%) and appointment reminders (6; 21%). The number of functions included in the interventions ranged from 1-6 (Table S5), with most interventions including three or fewer functions (22; 79%). Few studies referred to the use of a theoretical framework to design the intervention (5; 18%), although half acknowledged the involvement of people with HIV in their development (14; 50%). The majority of interventions (19; 68%) had been evaluated using either an RCT or pre-post study design.

### 3.4 SMS Interventions

#### 3.4.1 Overview of SMS Interventions

Of the 16 interventions delivered via SMS, 11 were designed to increase adherence to ART, 3 to increase engagement with care (*e.g.* to increase appointment attendance) and 2 to facilitate a range of self-management behaviours including adherence to ART, appointment attendance and lifestyle factors (complex self-management). These interventions are summarized in (Table S6). SMS messages were typically covert, without referring to HIV, antiretrovirals or potentially stigmatizing behaviours (*e.g.* recreational drug use). For example, several studies used vague messages such as “take ur big blue pill” [23] while others used more coded statements such as “Carry on! Carry on!” [24], “take good care of your health” [25] or “How are you?” [26]. In several instances, the content of the SMS message was personalized by allowing the individual participants to choose the message [23, 24, 27-29]. Seven of the SMS interventions (44%) employed one-way text messages, which predominantly served as a reminder. The remaining nine (56%) interventions employed 2-way text messaging, whereby participants could respond, either to confirm adherence behaviour (*e.g* [23, 27, 30].) or to access further support (e.g [26, 31].). In some cases, further SMS messages were then tailored according to the participant’s response. For example, tailored reinforcement messages were sent, such as “Great job! Ur current adherence: 75%. Adhr when u take ur next dose: 80% (4/5 doses)” [23] or the intervention intensity and content of subsequent messages was dynamically tailored to the individual’s needs [32-34]. For example, participants reporting substance use and potentially risky sexual behaviour could be sent tailored messages such as “Stay in control—guys who are buzzed or high take more risks” [33].

#### 3.4.2 Efficacy of SMS Interventions

Eight of the SMS interventions designed to improve medication adherence were evaluated in RCTs or pre-post trials. Seven were found to have a positive impact on adherence. In over half of these evaluation studies, objective adherence measures such as electronic monitoring or pharmacy refill rates were used. Four of the eight studies went on to explore the impact of the SMS intervention on clinical markers such as viral load and CD4 count, with two reporting a significant reduction in viral load [26, 34] and one reporting a significant increase in CD4 count [34] following the intervention. One of the studies that did not find a significant effect of the intervention on clinical markers had a very small sample size (n=25) and was unlikely to have been powered to find a difference [27].

Three of the SMS interventions designed to increase engagement with care were evaluated using an RCT [35, 36] or pre-post design [37]. Two of these interventions involved sending SMS appointment reminders and neither found a significant impact on HIV clinic appointment DNA rates [35, 37], although one study found a reduction in DNA rates among a larger general sexual health clinic population [37]. One study found that an SMS intervention alerting people to an abnormal CD4 result, combined with travel re-imbursement, significantly reduced the time to clinic return and the time to ART initiation [36].

Of the two SMS interventions designed to address a range of self-management behaviours [29, 33, 34], only one has been evaluated for effectiveness [33, 34]. In a relatively small pre-post trial (n=52), in addition to a positive impact on adherence, viral load and CD4 count outlined above [34], this tailored responsive SMS intervention was found to reduce reported sexual risk behaviours and increase perceived social support and HIV knowledge [33]. There was no significant change in participants’ perceived involvement in care, although this was already high before the initiation of the intervention.

#### 3.4.3 Feasibility and Acceptability of SMS Interventions

SMS interventions have generally been found to be feasible and acceptable to patients [25, 27, 28, 32, 38-40]. Benefits reported by participants include being reminded to take medicines, being reminded about appointments, having quicker/easier access to healthcare or psychosocial support and feeling cared for/ connected [25, 38, 39]. For example, one participant said “It was very helpful for me. Well sometimes I feel really alone, so it made me not feel so alone” [39]. However, in one study over 50% of participants found the text messages irritating [41] and participants in another study indicated that text messages would be unwelcome if they were too frequent [42]. Practical barriers to engaging with the SMS interventions included lost phones/ chargers and temporary service disconnection [28, 29, 35].

Two studies explored health care professionals’ perceptions of SMS interventions [43, 44]. Participants in both studies reported observed benefits for patients but also highlighted increased demands on their time (*e.g.* to respond to text messages) which would need to be taken into account in any roll-out of such interventions. There was some recognition, however, that the increased work load could be worthwhile due to adherent patients ultimately being less work – *e.g.* “if this is really successful and people do… start to adhere [to their HIV medication]…[the workload] would even out over time.” [43]. Some health professionals also reported concerns about face-to-face care being replaced with text messaging – *e.g.* “I would hate to see it as though this is how your care is now, is *via* texting, or phone, or whatever, versus in person” [43].

### 3.5 App Interventions

#### 3.5.1 Overview of App-Based Interventions

Five interventions delivered via smart device apps were identified, outlined in Table S7. Two were developed specifically with an aim of increasing medication adherence [45, 46]. Both were based on an app developed by Media Lab, Massachusetts Institute of Technology. The app enabled participants to record their medication-taking using a clock programmed with their medication schedule. It also provided visual information on adherence (*e.g.* though graphical representations of estimated plasma concentrations of antiretroviral medicines) and clinical information (*e.g.* viral load, CD4 count).

Two apps addressed multiple aspects of self-management [47-49]. One involved the use of an existing, low-priced Personal Health Record app (MyMedical), downloaded onto iPods [47], to enable participants to enter, store and retrieve information to support self-management (*e.g.* record test results and appointment details, set medication reminders). Another used an existing survey app (ohmage) to enable participants to monitor their medication adherence and risk behaviour (*e.g.* substance use, sexual behaviour), as well as mental health and stressful life events [48].

The final app was designed to reduce alcohol consumption among alcohol dependent people living with HIV [49]. The research team worked with android programmers and service users/ health professionals to develop a smart phone adaptation of a previous IVR intervention (HealthCall). Following a motivational interviewing session, participants used the app to record their daily alcohol intake, medication adherence and risk taking behaviour. They were also given access to a counsellor and could obtain graphical feedback on their drinking patterns.

#### 3.5.2 Efficacy of App-Based Interventions

Perera *et al.* (2014) conducted an RCT to determine the effectiveness of their smartphone adherence app [45]. The full intervention was associated with significant improvements in viral load, self-reported adherence and satisfaction compared to the active control condition, where patients only received the clock/ adherence-monitor element. Greater levels of engagement with the app were associated with increased understanding of HIV infection and perceived need for ART.

Luque *et al.* (2012) reported a significant increase in self-efficacy for adherence following the use of the personal health record application [47]. This appeared to be driven by increased self-efficacy for integrating medication into daily routines. However actual adherence behaviour was not assessed.

Hasin *et al.* (2014) reported a decrease in the number of drinks per day and an increase in 30-day abstention following the HealthCall-S app [49]. These findings were comparable with those achieved with the IVR version of the intervention.

#### 3.5.3 Feasibility and Acceptability of App-Based Interventions

The app-based interventions appeared to be both feasible and acceptable to participants. For example, 92% participants reported that they liked using HealthCall-S [49], 81% participants who used the personalized visual adherence app said they would recommend it to other people on ART [45] and over 90% of participants who used the personalized health record on the iPod were satisfied with the ease of use of both the app and the device, and intended to use it after subsequent clinic visits [47]. However, approximately a third of patients using HealthCall-S reported some concerns about privacy [49].

Swendeman *et al.* (2015) conducted semi-structured interviews with participants using the self-monitoring survey app [48]. Participants reported numerous benefits of self-monitoring, achieved primarily through mechanisms of reflection (*e.g.* “Helped me think about how many times I actually miss my medication—I never thought about it before”), reinforcement (*e.g.* “Helps me stay on track with not smoking”) and cues to action (*e.g.* “Reminds me to ask the questions about safe sex and find out the status of my partner”). The findings suggested that self-monitoring may influence beliefs, motivations and skills. However, almost a third of participants found the surveys tedious/ repetitive [48].

### 3.6 Mobile Phone Calls

#### 3.6.1 Overview of Mobile Phone Call Interventions

Three interventions used calls to a mobile phone to deliver self-management interventions. The first targeted medication adherence among young people living with HIV through daily calls by an adherence facilitator to address barriers to medication adherence, provide support for problem solving, make referrals to the clinical team where required and give appointment reminders [50, 51]. The second focused on medication adherence and appointment attendance among ART naïve and experienced patients in China, with two-weekly, 3-minute calls addressing difficulties in making hospital visits, treatment adherence and health concerns [52]. The final mobile phone call intervention addressed smoking behaviour [53]. Following routine smoking cessation advice and information on how to obtain nicotine replacement therapy patches, intervention participants received 11 cognitive-behavioural therapy (CBT)-based counselling sessions over 3 months, focusing on motivation to quit, self-efficacy and social support. They were also given access to a hotline to call if additional support was required between sessions. None of the phone call interventions specified user involvement in their development.

#### 3.6.2 Efficacy of Mobile Phone Call Interventions

Belzer *et al.* tested the adherence intervention in an RCT and found that the daily calls with an adherence facilitator was associated with a significant increase in self-reported adherence and a significant decrease in viral load [50]. In contrast, brief two-weekly calls did not have a significant impact on adherence, clinical attendance or clinical variables among either treatment-naïve or treatment experienced patients, although adherence was already high at baseline [52]. ART-naïve patients who received the intervention experienced a significant increase in some quality of life domains following the intervention

In an RCT of the smoking cessation intervention, the odds of 7-day abstinence were more than 4 times higher among participants who received the intervention than in the control group [53]. In a secondary analysis, self-efficacy was identified as a significant mediator of the intervention effect [54].

#### 3.6.3 Feasibility and Acceptability of Mobile Phone Call Interventions

Perceptions of mobile phone call interventions were only elicited from participants who received the adherence facilitator phone call intervention [51]. In exit interviews, 94% of participants interviewed gave very positive feedback, reporting that they felt supported by the adherence facilitator, and experienced increased in motivation and routine for taking ART following the intervention. Reminders and support were perceived to be important features of the intervention. 81% said they would have liked to have continued with the intervention and 100% reported that they would recommend the intervention to a friend. Adherence facilitators were also interviewed, with 92% believing that the participants were able to make use of the problem solving discussions.

### 3.7 Other Intervention Delivery Formats

Swendeman *et al.* (2015) worked with a community advisory board to develop and pilot a mobile IVR based intervention consisting of a series of recorded messages aimed at addressing multiple domains of self-management [55]. Calls were made daily corresponding to the individual’s ART regimen. There was a significant increase in adherence following the intervention, however, increases in depression and alcohol use (frequency of drinking) were also observed. Participants reported concerns about messages being overheard by others, however majority of messages had high approval rates. Participants preferred general health (rather than HIV-specific) messages, and valued messages addressing hygiene, nutrition, physical and psychological health/wellbeing and adherence reminders [55].

The HIV India (HIVIND) intervention utilised two types of weekly mobile phone adherence reminders (i) an automated interactive voice response (IVR) call and (ii) a non-interactive neutral picture SMS [56-58]. The acceptability of the intervention was examined in two studies. Over 90% reported that the intervention was useful [58] however the IVR component of the intervention was considered to be more helpful than the text message [56]. In a pre-post study, the proportion of patients with high adherence increased significantly following the intervention [56] however a subsequent RCT found no significant effect of the intervention on viral load, adherence or mortality in 631 patients initiating ART [57].

Winstead-Derlega (2012) examined the feasibility and potential impact of delivering peer-health messages on HIV-medication adherence, stigma and disclosure, via videos pre-programmed onto an iPod Touch [59]. The majority of participants reported that the videos were helpful and meaningful and were interested in viewing more, however, there were no significant changes in questionnaire items measuring engagement with care or comfort with HIV disclosure between baseline and follow-up [59].

Stankievich (2015) evaluated an intervention involving twice-monthly contact with healthcare professionals through text message or a social networking site of the participant’s choice (*e.g.* Facebook, WhatsApp), and a mobile-phone contact if needed [60]. The intervention was considered to be feasible and was associated with improved adherence in a group of children and young adults.

## 3.8 Protocols

Four published protocols were identified (Table S2). All described an SMS intervention. Of these, one intervention focused on adherence to ART through providing daily medication reminders [61]; two focused on engagement with care (providing adherence reminders plus supportive, informational and motivational text message [62] or weekly two-way SMS to check how patients are doing and allow them to identify whether assistance is required [63]), and one intervention focused on both adherence and engagement with care, comprising text messages related to appointment reminders; medication adherence, HIV secondary prevention and barriers to care [64].

## 3.9 Currently Funded Studies

Five further current studies were found on ClinicalTrials.gov (Table S3). These included four SMS interventions and two apps (1 intervention included both SMS and an app). Three of the SMS interventions focused on adherence to ART (two of which provided feedback on adherence and one of which provided adherence reminders with monetary reinforcement for adherence). One intervention was focused on both engagement with care and adherence, providing appointment reminders as well as medication reminders.

## 4 DISCUSSION

We identified 41 research papers published since 2012, reporting on 28 mHealth self-management interventions to support individuals receiving HIV treatment and care. Most of the mHealth interventions reviewed and currently in development were SMS-based. Other formats included apps, mobile phone calls, interactive voice response and downloaded videos. No single format stood out above the others in terms of effectiveness, feasibility or acceptability.

The findings of this systematic review suggest that there has been progress in the development and evaluation of mHealth interventions for PLWH over recent years. In an earlier systematic review, Catalani *et al.* (2013) identified 35 research studies published in the years up to 2011 reporting on the development or evaluation of mHealth interventions addressing HIV prevention, care and treatment [17]. The investigators noted the preponderance of small scale, early stage studies exploring feasibility and acceptability of mHealth interventions and only a small number of impact evaluations. In contrast, two thirds of the interventions included in our review had been evaluated using a randomized controlled trial or prospective, repeated measures design.

Our review found evidence that mHealth interventions can have significant impact on outcomes including adherence to ART [23, 24, 26, 27, 30, 34, 45, 55], viral load [26, 34, 45], engagement with care [36], HIV knowledge, social support and sexual risk behaviours [33], smoking cessation [53], quality of life [52], depression, anxiety and self-efficacy [54]. However, none of the studies identified evaluated the cost-effectiveness of mHealth interventions, which may be necessary in order to convince health care providers of the value of wide-scale implementation of mHealth interventions as a viable addition to clinical care.

The importance of an implementation sciences perspective in the development, evaluation and implementation of mHealth interventions has been widely acknowledged [11, 13, 17]. Members of target populations were involved in the development of 50% of the interventions included in this review. However the degree to which PLWH were involved differed between studies, from the use of focus groups to inform the development of an application [46] to the iterative testing and development of the intervention [47]. A recent study identified the desired functions of mHealth self-management apps among a group of PLWH [15]. These included functions addressing patient-provider and peer communication; medication and appointment reminders; a medication checklist; lab reports; pharmacy information; nutrition and fitness trackers; resources (such as social services, substance abuse, video testimonials) and search and settings functions. None of the interventions identified in our review included all of these functions. More than half of the interventions included medication reminders and over a third included patient-provider communication. Few, however, included lab reports and links to resources and no interventions included nutrition or fitness trackers. Interventions varied widely in the number of functions they offered, with 29% of interventions comprising a single function (typically a medication reminder) and only 22% of interventions including four or more functions. There is clearly a need to develop more complex apps in line with patients’ needs. An obvious challenge is the cost of developing this technology. Rather than individual research groups building apps in isolation, there may be a need for greater collaboration or indeed to explore opportunities to partner with commercial organisations.

Patients reported satisfaction with the interventions [27, 32, 49] and many participants planned to continue with the intervention after the study finished [27, 47, 51] or said they would recommend the intervention to a friend [42, 45, 51]. Although participants in most studies found medication reminders helpful [25, 42, 58], more than half the participants in one study found the medication reminder messages annoying [41], indicating the need for user involvement in the development of interventions.

Potential barriers to uptake, engagement and persistence with interventions were also identified. Privacy concerns associated with the interventions were reported by a proportion of participants (ranging from 5-38%) [42, 49, 58]. The majority of the interventions reviewed (54%) offered covert features and messages in order to protect confidentiality. Barriers to engagement with interventions included lost phones/chargers and temporary disconnection [28, 29, 35]. This highlights a potential barrier to persistence with mHealth interventions when implemented into care programmes, especially given that patients were provided with phones and/or received reimbursement for intervention costs in 50% of the studies. Healthcare professionals reported benefits of mHealth interventions including improved patient care and were interested in upscaling the intervention, however they had concerns about the increased demands on staff time associated with implementation of the intervention into care [44]; and the potential replacement of valuable face to face care with text messages [43].

### 4.1 Limitations

Only a small proportion of the interventions were developed using a theoretical framework. In relation to adherence, the National Institute for Health and Care Excellence (NICE) [65] recommends a perceptions and practicalities approach, suggesting that interventions will be more effective if they address both perceptual barriers (such as individuals’ perception of their personal necessity for treatment and concerns about potential adverse effects) as well as practical barriers (such as forgetting). While the interventions included in this review addressed practical barriers through medication reminders and behavioural monitoring, few targeted perceptual barriers. mHealth interventions may benefit from incorporating components which tailor messages to individual’s personal perceptions of HIV and ART. An intervention designed to improve adherence to corticosteroids for asthma found that an individually tailored text messages reduced threatening illness perceptions and increased perceived necessity for treatment and adherence [66].

Participants were provided with incentives for using the mHealth intervention (*e.g.* money/ gift tokens/ phones/ tariff) in 50% of the studies reviewed. This was particularly the case when trying to reach particularly high risk or vulnerable groups (*i.e.* those least engaged with healthcare/ low adherers). It is arguable that the findings will not therefore be generalizable to routine clinical practice where such incentives are unlikely to be provided/ cost-effective. One avenue for future research could be the introduction of gamification strategies (*i.e.* using game type elements such as digital rewards (*e.g.* points) and levels) that could make engagement with the apps intrinsically rewarding. Research suggests that such strategies are already employed regularly in commercial health and fitness apps [67]. Another under-researched area is the use of mHealth for PLWH who are stable on treatment and for whom use of a mHealth tool may facilitate self-management, preventing unnecessary routine clinic visits.

Our review was restricted to the research literature, however, there may be commercially available applications that are acceptable and beneficial to patients but which have not been included in this review. Negative findings may not be published, therefore the review may not be comprehensive. Furthermore, we did not include generic mHealth self-management interventions unless they had been specifically evaluated with people living with HIV. It may be that there are applications that are useful to PLWH but which have not yet been evaluated within this group.

## CONCLUSION

Over the past few years, there has been an increase in the number of self-management mHealth interventions evaluated with PLWH. These interventions have been shown to be effective, feasible and acceptable. However, the literature is dominated by interventions offering a small number of limited functions, such as medication reminders. mHealth has the potential to offer patients a much wider range of functions, such as opportunities to monitor and track clinical markers of disease progression, symptoms, nutrition and exercise, and to provide access to tailored information, educational messages and online peer support, depending on the needs and preferences of patients. With predicted rises in the use of health related applications over coming years, there is a need to develop, implement and evaluate more comprehensive mHealth interventions in order to address the self-management needs of people living with HIV.

## Figures and Tables

**Fig. (1):**
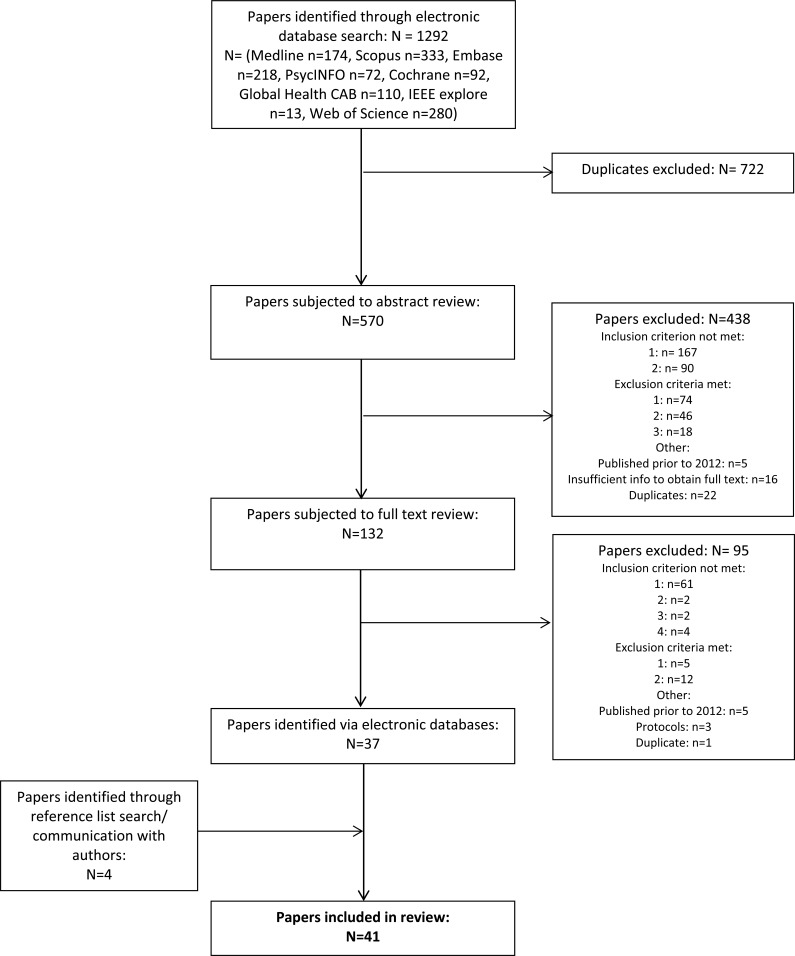
Flow diagram showing paper selection process.
