# Supplementary material for: mHealth Interventions To Support Self-Management In HIV: A Systematic Review
Source: Open AIDS J. 2017 Nov 21;11:119–32. doi: 10.2174/1874613601711010119 (PMC5730953; doi:10.2174/1874613601711010119)
Supplement: Supplementary file 1 [file TOAIDJ-11-119_SD1.pdf]

Send Orders for Reprints to reprints@benthamscience.ae

The Open AIDS Journal, 2017, *11*, i-xxxiii

BENTHAM OPEN

CrossMark

The Open AIDS Journal

Supplementary Material

Content list available at: www.benthamopen.com/TOAIDJ/

DOI: 10.2174/1874613601711010119

mHealth Interventions To Support Self-Management In HIV: A Systematic Review

Vanessa Cooper, Jane Clatworthy, Jennifer Whetham* and EmERGE Consortium

The Lawson Unit, Brighton and Sussex University Hospitals NHS Trust, Brighton, England

Supplementary Table 1. Characteristics of included studies (n=41).

**Reference/ Intervention**	**Country**	**Aim**	**Methods/ Design**	**mHealth element**	**Population (sample size)**	**Outcomes and measures**	**Findings**	**Quality assessment (MMAT)**
Belzer (2014) [50] **Cell phone Adherence Facilitator (AF) intervention**	USA	To determine if daily cell phone conversations with Adherence Facilitators around self-care and taking HIV medications would have an impact on ART adherence and viral load.	Quantitative RCT	Daily phone calls	HIV positive young people aged 15-24 with a history of ART non-adherence (n=37)	• Adherence to ART (visual analogue scale); viral load	• Significant effect of the intervention on adherence<br>• log ^{10} HIV VL significantly lower in intervention group at both 24 weeks and 48 weeks.	75%
Belzer (2015) [51] **Cell phone Adherence Facilitator (AF) intervention**	USA	To describe the acceptability and feasibility of a cell phone support intervention.	Quantitative Cross sectional	Daily phone calls	HIV positive young people aged 15-24 with a history of ART non-adherence (n=37)	• Feasibility: intervention and study retention rates.<br>• Acceptability: Content analysis of participant exit interviews assessing the acceptability of calls, feelings about the impact and suggestions to improve the intervention.	• Feasibility: 37% of participants discontinued the intervention (missed >20% of calls for 2 consecutive months or missed 10 consecutive calls).<br>• Acceptability: Patients reported it was easy to talk to adherence facilitators; found the reminders helpful, and found that getting calls made it easier to adhere to their medication. Most reported improved motivation, stating that the facilitator was supportive. All would recommend the intervention to peers.	25%

Suppl. Table 1 contd.....

Reference/ Intervention	Country	Aim	Methods/ Design	mHealth element	Population (sample size)	Outcomes and measures	Findings	Quality assessment (MMAT)
							• Cost was estimated to be approximately £500 per patient for a six-month intervention.	
Da Costa (2012) [25] **The HIV Alert System (HIVAS)**	Brazil	To assess whether a warning system based on mobile SMS messages increases the adherence of HIV-infected Brazilian women to antiretroviral drug-based treatment regimens and their impressions and satisfaction with respect to incoming messages.	Quantitative RCT	SMS	HIV positive women (n=21)	• Adherence (self-report, pill count and microelectronic monitoring) • Satisfaction (5 questions responses on likert scales)	• A greater percentage of patients in the intervention group than in the control group were adherent (self-report, pill count and electronic monitoring) but these differences were not statistically significant (NB low power due to small sample size). • Patients were satisfied with the messages received, which were seen as reminders, incentives and signs of affection by the health clinic for a marginalized population.	50%
Dowshen (2012) [27] **Intelecare**	USA	To evaluate the feasibility, acceptability, and preliminary efficacy of short message service (SMS) or text message reminders to improve adherence to ART for youth living with HIV/AIDS.	Quantitative Pre-post	SMS	HIV positive, age 14-29, on ART with low adherence (n=25)	• Adherence (visual analogue scale and adherence surveys from the AIDS Clinical Trials Group) • Feasibility • Acceptability • Viral load • CD4 count	• Significant increase in adherence compared to baseline. • Enrolment and retention of the study population was feasible with 25 participants enrolled over a 6-month period 92% and 84% of participants completing all visits at 12 and 24 weeks, respectively. • High satisfaction: 81% participants would like to continue to receive text messages after the end of the study, 95% participants indicated that the text messages helped them “very much” to miss fewer doses of medication.	75%

Suppl. Table 1 contd.....

**Reference/ Intervention**	**Country**	**Aim**	**Methods/ Design**	**mHealth element**	**Population (sample size)**	**Outcomes and measures**	**Findings**	**Quality assessment (MMAT)**
							• No significant change in viral load or CD4 count (trend towards improvement).	
Farmer (2014) [37]	UK	To measure the impact of text reminders on reducing the non-attendance rates for patients attending sexual health and HIV clinics.	Quantitative Retrospective pre-post study	SMS	Patients attending pre-booked sexual health and HIV appointments	• Did not attend (DNA) and cancellation rates in sexual health and HIV appointments over two 12 month periods (before and after introducing SMS reminder service)	• 4% decrease in DNA rates overall but no significant change in HIV clinic DNA or cancellation rates.	100%
Furberg (2012) [32]	USA	To develop, implement, and test a tailored SMS-based intervention for HIV-positive MSM.	Quantitative Pre-post	SMS	HIV positive MSM (n=52)	• SMS response rates. • Participants' perceptions of the content, timing, volume, usefulness of the messages.	• 92% responded to at least one of the weekly medication adherence questions administered via SMS. • Of those who responded to questions about the SMS messages, 93% indicated that they always read the text messages and approximately 75% said that they liked the messages. • 50% said they were always or usually delivered at the right time. • 62% said that the number of messages was "about right." • About half indicated that the message topics were either somewhat or very interesting to them. • More than two-thirds said the text messages were either somewhat or very helpful.	75%
Hailey (2013) [30] **STAR TRACK Adherence to Medication Program (STAMP)**	USA	To increase the proportion of youth who were adherent to ART, and increase the number of youth retained in HIV medical care and support services	Quantitative Retrospective comparison	SMS	HIV positive adolescents and young adults (age 13-24) (n=87)	• Details not provided	• All but 1 patient remained in Stage 1 • Self-reported adherence increased from approximately 40-50% (pre ST-AMP) to approximately 80%.	25%

Suppl. Table 1 contd.....

Reference/ Intervention	Country	Aim	Methods/ Design	mHealth element	Population (sample size)	Outcomes and measures	Findings	Quality assessment (MMAT)
							• Adherence to medical appointments increased.<br>• Patients proactively communicated with staff via text (*e.g.* notified staff of conflicts with scheduled appointments, sought medical advice for minor concerns).	
Hasin (2014) [49] **HealthCall-S**	USA	To adapt HealthCall for delivery via on the smartphone (HealthCall-S) among alcohol dependent patients in HIV primary care, and determine HealthCall-S feasibility.	Quantitative Multiple measurement with historical control group	App	HIV positive, alcohol dependent adults (n=41)	• Mean number of drinks per drinking day in the prior 30 days, calculated using time line follow backs (TLFB).<br>• Patient perceptions and satisfaction measured using Likert-type items.	• Patients used HealthCall on the smart phone platform more regularly (85% of days) than those in the comparison group with the IVR version (64% days).<br>• Mean number of drinks per drinking day in the prior 30 days at baseline was 9.3 (intervention group) dropping to 3.9 at 60 days, similar to the reduction in the comparison IVR group (8.1 at baseline, 3.5 at 60 days).<br>• Percent days abstinent (PDA) in patients receiving the intervention increased from 58% at baseline to 79% at 60 days.<br>• Over 90% patients reported that they liked using Health-Call-S with the majority reporting that it reminded them of their drinking goal and initial meeting with their counsellor.<br>• 38% patients had some concerns about privacy.	100%

Suppl. Table 1 contd.....

Reference/ Intervention	Country	Aim	Methods/ Design	mHealth element	Population (sample size)	Outcomes and measures	Findings	Quality assessment (MMAT)
Huang (2013) [52] **Phone call intervention**	China	To investigate the effects of a phone call intervention on adherence to ART and quality of life (QOL) of treatment-naive and treatment-experienced patients.	Quantitative RCT	Phone call	HIV positive adults on ART (treatment naïve and treatment experienced groups) (n=196)	• Adherence to ART (self-report). • Follow-up rate • Clinical variables (CD4 count, weight change, opportunistic infections, WHO clinical staging) • QOL (WHOQOL-HIV brief).	• No statistically significant differences in adherence, follow-up rate or clinical variables between the intervention and control groups in either treatment-naïve or treatment experienced patients. • Among treatment naïve patients, those in the intervention group experienced a significantly greater increase in quality of life over the 3 months of the study than those in the control group.	75%
Ingersoll (2014) [29] **Treatment Extension by Text (TxText)**	USA	To develop and assess feasibility/ acceptability of a two-way text messaging tool to address ART adherence, depressed mood, and active substance use.	Quantitative RCT (reporting process data from intervention arm only)	SMS.	Non-adherent HIV positive substance users (n=31 in intervention arm).	• Process outcomes (number of messages sent and received, query type, rates of non-response).	• Participants responded to 69% of the medication queries, 67% of the substance abuse queries and 64% of the mood queries. • 19 instances of nonresponse periods ranging from 7 - 23 days (mean 13.9 days (SD= 5.8). • Reasons included hospitalization, incarceration, lost, stolen, or damaged phones, and lost chargers.	50%
La Grange (2012) [28] **CareSpeak**	USA	To explore how a research team introduced a novel adherence tool to a group of 16-22 year olds during a 30-day feasibility study.	Qualitative and quantitative Cross-sectional	SMS	HIV positive youth (sample size not stated)	• Participants assessed the usability, impact on their lifestyle and privacy and whether they felt that the program was helpful in their daily medication taking regimen.	• Participants felt the SMS program did not interrupt their lifestyle or daily activities. • Privacy was not an issue as most individuals reported that they were the only ones that looked at their text messages. • Challenges included problems receiving and responding to text messages, and cell phone service temporarily disconnected.	NA (conference abstract)

Suppl. Table 1 contd.....

Reference/ Intervention	Country	Aim	Methods/ Design	mHealth element	Population (sample size)	Outcomes and measures	Findings	Quality assessment (MMAT)
Lewis (2013) [34] **Tailored text messages**	USA	To determine whether dynamically tailored medication messages delivered to people living with HIV (PLWH) via text messaging would be well received and enhance adherence and clinical outcomes	Quantitative Pre-post	SMS	HIV positive MSM (n=52)	• Self-reported adherence ("Over the past 7 days, on how many days did you miss a dose of medication?").<br>• Reasons for missing medication doses (checklist).<br>• CD4 count<br>• Viral load<br>• Satisfaction	• The number of missed medication days reduced significantly for those who were non-adherent at baseline.<br>• Significant reduction in viral load and increase in CD4 from baseline to follow-up<br>• Greatest improvements were for participants who began the study with higher viral loads and lower CD4 counts.<br>• 39% and 29% reported the messages being "very" or "somewhat" helpful, respectively.	75%
Luque (2013) [47] **MyMedical**	USA	To assess usability, acceptability and impact on adherence self-efficacy of a personal health record (PHR) on a handheld device (iPod Touch)	Quantitative Pre-post	App	HIV positive adults (29)	• Perceived self-efficacy for HIV (HIV Treatment Adherence Self-Efficacy scale)<br>• Assessments of device and PHR usability (7-point Likert-type scales).	• Improved self-efficacy for treatment adherence, driven by self-efficacy for integration of medication taking into one's routine.<br>• Preference for the device calendar to track appointments and the PHR for medication reminders.<br>• > 90% reported satisfaction with the ease of use of the iPod and the PHR.<br>• 100% of participants planned to use the PHR during or after their doctor visits.	75%
Mbuagbaw (2013) [68] **Cameroon Mobile Phone SMS (CAMPS)**	Cameroon	To describe the responses from the participants in the Cameroon Mobile Phone SMS (CAMPS) trial and the implications for health service providers	Quantitative Cross-sectional analysis of intervention arm of RCT	SMS	HIV positive adults (n=101 in intervention arm)	• Content of participant's responses to SMS grouped them into categories.	• Of 101 participants, 48 (47.5%) responded >= once by phone call or text message.	100%

Suppl. Table 1 contd.....

Reference/ Intervention	Country	Aim	Methods/ Design	mHealth element	Population (sample size)	Outcomes and measures	Findings	Quality assessment (MMAT)
							• 154 responses (99 phone calls and 55 text messages) were received over 6M.<br>• Half (n = 79) were expressions of gratitude.<br>• Other messages requested logistical support (n=21), required medical help (n=20), wanted to verify authenticity of text messages (n=9), requested counselling for HIV testing (n=2), requested financial help (n=1) or reported practical issues such as lost phones/ requests to withdraw from study (n=12).	
Mbuagbaw (2012) [31] **Cameroon Mobile Phone SMS (CAMPS)**	Cameroon	To evaluate the utility of weekly motivational SMS texts on improving adherence and other important outcomes among a representative sample of HIV-positive adults in Cameroon	Quantitative RCT	SMS	HIV positive adults (n=200)	• Adherence: Visual Analogue Scale (VAS); Self Report (SR); and Pharmacy Refill Data (PRD).<br>• Clinical outcomes: weight, body mass index (BMI), opportunistic infections (OI)<br>• QOL (SF-12)<br>• All-cause mortality<br>• Retention in the trial	• At 6 months, there was no significant overall effect on adherence by VAS, reported missed doses or number of pharmacy refills.<br>• No significant effect of the intervention on presence of a new opportunistic infection, mortality, retention in the trial, weight, Body Mass Index, Quality of life or CD4 count.	100%
Mbuagbaw (2012) [44] **Cameroon Mobile Phone SMS (CAMPS)**	Cameroon	To explore health workers' views on the use of text messages to improve adherence to ART	Quantitative Cross-sectional	SMS	Staff of the HIV clinic running the CAMPS trial (n=29)	• Questionnaire assessing views about the CAMPS trial.	• 69% reported observing benefits to patient care (*e.g.* improved medication adherence and appointment attendance).<br>• 3% reported negative effects (*e.g.* reduced adherence/ attendance).<br>• 62% felt that the intervention increased their workload.<br>• 76% would like to participate in the up-scaling of the intervention.	NA (conference abstract)

Suppl. Table 1 contd.....

Reference/ Intervention	Country	Aim	Methods/ Design	mHealth element	Population (sample size)	Outcomes and measures	Findings	Quality assessment (MMAT)
Moore (2015) [23] **Individualized texting for adherence building (iTAB)**	USA	To compare individualized texting for adherence building (iTAB) vs. active control (CTRL) on antiretroviral (ARV) and psychotropic (PSY) medication adherence among HIV-infected persons with co-occurring bipolar disorder (HIV+/BD+)	Quantitative RCT	SMS	HIV+ /bipolar disorder (BD+) patients (n=50)	• Adherence to psychotropic medicines and ART (electronic monitoring and self-report using visual analogue scale (VAS)	• Participants responded to over 90% of the mood and adherence text messages. • No significant differences between groups in mean adherence, as assessed by MEMS. • iTAB participants took ARVs significantly closer to their intended dosing time than control participants. • There was no group difference on psychotropic dose timing.	75%
Murray (2015) [26] **WelTel Oaktree**	Canada	To examine effectiveness of this weekly interactive text-messaging intervention (WelTel Oaktree) on improving ART adherence, VL, CD4 count and appointment attendance in British Columbia	Quantitative Pre-post	SMS	HIV+ participants, qualify for ART; with CD4<500, HIV VL >200 copies/mL and considered 'vulnerable' by the clinical team (*e.g.* unstable housing, active addiction, domestic violence, poor care engagement or adherence, advanced HIV infection/AIDS, or mental health factors) (n=81)	• Adherence (pharmacy refill records and patient reports) • CD4 count • Viral Load • Appointment attendance.	• Population VL decreased significantly • ART adherence improved significantly • No significant change in CD4 count or appointment attendance.	NA (conference abstract)
Murray (2015) [43] **WelTel Oaktree**	Canada	To contextualize HCP experiences in context of the patient-provider interactions over the course of the intervention, then to explore the experiences of HCPs as they pertain to acceptability and feasibility of the WelTel mHealth intervention.	Quantitative and qualitative Cross-sectional	SMS	HIV positive patients from five demographic/ 'risk' categories (youth, mature, English as a second language, remote and Low CD4/ high VL) (n=25)	• SMS data; thematic analysis of focus group and interview transcripts	• On average 56.9% of participants responded to the text message each week. • On average, 15.2% of participants indicated a problem each week.	50%

Suppl. Table 1 contd.....

Reference/ Intervention	Country	Aim	Methods/ Design	mHealth element	Population (sample size)	Outcomes and measures	Findings	Quality assessment (MMAT)
							• Baseline focus group with HCPs highlighted the importance of recognizing the Oak Tree clinic model of care, anticipated benefits for patients, and concerns about replacing face-to-face care with texting and the burden on HCPs' time.<br>• Results of study-end interviews with HCPs involved with the study revealed two main themes believed important to clinical practice: (1) the workload required to integrate WelTel into the Oak Tree model of care, and (2) the usefulness of the intervention to engage, empower, and interact with patients.	
Murray (2013) [69]<br>**WelTel Oaktree**	Canada	To assess the feasibility and acceptability of a weekly interactive text-messaging intervention using the WelTel model in HIV+ individuals taking HAART in British Colombia	Qualitative and quantitative Pre-post	SMS	HIV positive patients from 5 groups ('youth', 'mature', 'English as a second language', 'remote', and 'CD4 <200') (n=25)	• Semi-structured qualitative interviews/ questionnaires subjected to thematic analysis.<br>• Demographics, CD4s, viral load, physician recorded adherence, and weekly responses were collected.	• The intervention was perceived to be feasible, acceptable, and an improvement in care.<br>• Of 20 exit interviews completed, all would recommend the intervention to a peer.<br>• Mean HIV VL declined by 0.28 log, mean CD4 increased by 53 cells/mL and adherence increased in 29% of participants over the 6-months.	NA (conference abstract)

Suppl. Table 1 contd.....

Reference/ Intervention	Country	Aim	Methods/ Design	mHealth element	Population (sample size)	Outcomes and measures	Findings	Quality assessment (MMAT)
Norton (2014) [35] **Call-em-All (online texting service)**	USA	To determine the acceptability and feasibility of text message appointment reminders among persons living with HIV in the USA.	Quantitative RCT	SMS	HIV positive adults (n=52)	• Appointment attendance.<br>• Feasibility outcomes: uptake and use of the intervention.	• No difference in attendance between intervention and control group.<br>• Of 94 screened, 52 (55%) elected to participate.<br>• 11/25 (44%) did not correctly opt-in to the commercial texting service and were sent reminder texts manually by the investigator.<br>• 24% (6/25) had mobile phones disconnected during the study.	100%
Perera (2014) [45] **Smartphone application incorporating personalized health-related visual imagery**	New Zealand	To examine the efficacy of a smartphone application incorporating personalized health-related visual imagery that provided real-time information about the level of medication and the current level of immunoprotection in the patient's body, in order to facilitate adherence to ART.	Quantitative RCT	App	HIV positive patients on ART for at least 6 months; used an Android smartphone (n=28)	• Adherence based on self-report (Medication Adherence Report Scale and % doses taken), pharmacy dispensing records and viral load<br>• Illness perceptions (Brief Illness Perceptions Questionnaire).<br>• Beliefs about treatment (necessity and concerns).<br>• Google Analytics software employed to determine use of the app.<br>• Questionnaire on perceptions of the app.	• Those who received the enhanced app had higher adherence and lower viral load at three months than those in the control group.<br>• Greater levels of engagement with the app were associated with greater understanding of HIV infection and increased perceived need for ART.<br>• 81% of intervention participants would recommend the app.<br>• 79% indicated that the addition of an alarm function would be useful.	50%
Reid (2014) [42] **SMS reminders**	Botswana	To establish whether using SMS reminders for medication refills was acceptable to patients on ART.	Quantitative Cross-sectional data from an RCT	SMS	HIV positive adults enrolled in an RCT evaluating the impact of SMS reminders on ART pharmacy collection and clinical outcomes (n=42 in intervention arm)	• Acceptability of SMS reminders as an adherence intervention<br>• Perceived barriers to SMS reminders in HIV care<br>• Perspectives on the role of SMS interventions across the spectrum of HIV services.	• 93% stated that the SMS reminders were somewhat or very helpful.<br>• 10% expressed concern about inadvertent HIV status disclosure.<br>• Only 20% stated that they would want to receive daily SMS pill reminders.<br>• 79% would recommend the SMS service to others who have HIV.	100%

Suppl. Table 1 contd.....

Reference/ Intervention	Country	Aim	Methods/ Design	mHealth element	Population (sample size)	Outcomes and measures	Findings	Quality assessment (MMAT)
Rodrigues (2012) [56] **HIVIND**	India	To assess the influence of mobile phone reminders on adherence to antiretroviral therapy in South India and to explore patients' experiences with the intervention.	Quantitative Pre-post	SMS/IVR	HIV positive adults on ART (n=150)	• Adherence (pill count) • Barriers to adherence (ACTG barriers to adherence self-report). • Participant experiences assessed on a five-point Likert scale.	• The proportion of participants with optimal adherence (≥95%) increased from 85% to 91% during the intervention period. This effect was maintained 6 months after intervention. • Both IVR calls and SMS reminders were considered non-intrusive and not a threat to privacy. • A significantly higher proportion agreed that the IVR was helpful compared to the SMS.	100%
Rosen (2015) [46]	USA	To adapt and develop an mHealth app for HIV patients with histories of substance abuse.	Qualitative Cross-sectional	App	HIV positive patients with history of substance abuse (n=22)	• Participant responses to candidate app characteristics and their understanding of the HIV disease state based on these changing images.	• A balance of provided and requested information is important to maintain interest and support adherence. • App characteristics and information can provoke positive and negative reactions and these emotional responses may affect adherence.	NA (conference abstract)
Sabin (2015) [24] **China Adherence through Technology Study**	China	To assess the effect of a real-time feedback using triggered cell phone reminders coupled with Wisepill-generated data-enhanced counselling.	Quantitative RCT	SMS	HIV positive adults considered at risk of non-adherence (n = 119)	• Adherence assessed through electronic monitoring (WisePill). • CD4 count • UD viral load	• Those who received the intervention were significantly more likely to achieve optimal adherence (>95%) than those in the control group. • No significant differences in VL and CD4 count between intervention and control groups.	100%
Shet (2014) [57] **HIVIND**	India	To assess whether customised mobile phone reminders would improve adherence to therapy and thus decrease virological failure among HIV infected patients starting antiretroviral treatment (ART).	Quantitative RCT	SMS/IVR	ART naïve, HIV positive adults eligible to initiate first-line ART (n=631)	• Time to virological failure (plasma viral load >400 copies/mL on two consecutive samples measured at least one month apart)	• No significant differences in time to virological failure, adherence, death or attrition rates between groups.	100%

Suppl. Table 1 contd.....

**Reference/ Intervention**	**Country**	**Aim**	**Methods/ Design**	**mHealth element**	**Population (sample size)**	**Outcomes and measures**	**Findings**	**Quality assessment (MMAT)**
						• Adherence to ART (pill count)<br>• Attrition rates<br>• Death rates		
Sidney (2012) [58]<br>**HIVIND**	India	To assess the perceived usefulness and acceptability of mobile phone reminders to support adherence to ART	Quantitative Cross-sectional	SMS/IVR	HIV positive adults, on ART, mobile phone access (n=139)	• Participants' experience and perceived usefulness of the intervention was assessed through structured questionnaire at 1 month.<br>• Data on the delivery of the intervention were also obtained from an online database that recorded all outgoing IVRs and SMSs	• 76% of calls were received by the participants.<br>• 59% of participants viewed all the SMSs that were delivered, while 15% viewed none.<br>• Almost 90% of participants reported the intervention as being helpful as medication reminders<br>• Less than 5% felt the intervention was an intrusion on their privacy but 20%/ 13%, reporting that another person had inadvertently received their reminder call / SMS respectively.	100%
Siedner (2014) [36]	Uganda	To explore the effectiveness of a an SMS intervention to improve linkage to HIV care	Quantitative RCT	SMS	HIV positive adults, mobile phone access (n=553)	• Time to return to clinic after an abnormal result<br>• Time to initiation of ART among the ART naïve.	• Among those with an abnormal test results, those in the SMS group returned to the clinic and initiated ART significantly earlier than those in the control group.<br>• Among those with normal results, those who received an SMS message were significantly more likely to return to the clinic within 7 days of their scheduled return date.	NA (conference abstract)
Siedner (2015) [70]	Uganda		Quantitative Secondary analysis of data from an RCT	SMS	HIV positive adults, mobile phone access (n=385)	• Date of return to the clinic<br>• Questionnaire/ interview assessing whether or not participants received the message,	• 72% successfully received a message<br>• 87.6% correctly identified the message format<br>• 60.8% returned to clinic at the requested time.<br>• Among participants with abnormal test results (35.8%),	75%

Suppl. Table 1 contd.....

Reference/ Intervention	Country	Aim	Methods/ Design	mHealth element	Population (sample size)	Outcomes and measures	Findings	Quality assessment (MMAT)
		To identify predictors of uptake of an mHealth application for a low-literacy population of people living with HIV (PLWH) in rural Uganda and to evaluate the efficacy of various short message service (SMS) text message formats to optimize the balance between confidentiality and accessibility.				the number of SMS text messages received, and identification of the type of message received (direct, PIN-protected, coded).	the strongest predictors of reported message receipt were the ability to read a complete sentence and ability to access a test message on enrolment.<br>• No differences in outcomes between those receiving direct or coded messages.<br>• Those who were sent a PIN-protected message were less likely to return to the clinic within 7 days.	
Smillie (2014) [39]<br>**Wel-Tel Oaktree**	Canada	To understand the attitudes of people living with HIV who faced multiple barriers to engagement in HIV care, and their experiences using the Wel-Tel intervention as a tool to help manage their care.	Qualitative Pre-post	SMS	HIV positive participants - youth; mature; English as a second language, remote and non-suppressed. (n=25)	• Interview topics included acceptability of text messaging to communicate with HCP, optimal strategies for using text messaging, barriers to adherence and suggestions for reducing barriers, and ideas to improve the intervention.	• Participants described the intervention as a useful way to communicate with health care providers, thus increasing the ability to access services, report side-effects, and attend appointments.	75%
Stankievich (2015) [60]<br>**Mobile communication intervention**	Argentina	To evaluate the effects of a communication strategy using mobile devices on adherence to ARV treatment.	Quantitative Pre-post	Generic apps/ SMS/ phone calls	HIV positive young people (aged 6-25yrs) with suboptimal adherence (n=22)	• Viral load measured before and after the intervention as a proxy for adherence to ART.	• Ten (45%) preferred to be contacted by WhatsApp, 8 (36%) by text message, 4 (18%) by Facebook and others.<br>• 65% of the contacts generated additional requests about medications, appointments or symptoms.<br>• After eight months of the intervention, 20/22 VL results were available. 13/20 (65%) were undetectable, 14/20 (70%) had VL <1000 copies/ml. Six of twenty (30%) VL had no changes.	NA (conference abstract)

Suppl. Table 1 contd.....

Reference/ Intervention	Country	Aim	Methods/ Design	mHealth element	Population (sample size)	Outcomes and measures	Findings	Quality assessment (MMAT)
Swendeman (2015) [48] **Smartphone self-monitoring**	USA	To elaborate a theoretical model for the potential benefits of self-monitoring in supporting self-management of medication adherence, mental health, substance use, and sexual risk behaviours and to explore potential differences in efficacy of daily versus biweekly self-monitoring, barriers and challenges.	Qualitative Cross-sectional analysis of an RCT	App	HIV positive patients who used alcohol, tobacco, or other drug (ATOD) and engaged in sexual activity (at least once/week) (n=50)	• Qualitative analyses examine biweekly open-ended user-experience interviews regarding perceived benefits and barriers of self-monitoring, and to elaborate a theoretical model for potential efficacy of self-monitoring to support self-management for multiple domains	• About a third of participants reported that surveys were too long, frequent, or tedious.<br>• Daily self-monitoring was perceived by some as more beneficial than biweekly.<br>• About twice as many daily self-monitoring group participants reported increased awareness and behaviour change support from self-monitoring compared with biweekly web-survey only participants.	50%
Swendeman (2015) [55] **IVR with multiple domains**	India	To examine the potential of daily IVR messaging for ART adherence.	Quantitative and qualitative Pre-post	IVR	HIV positive adults (n=46)	• AIDS Clinical Trial Group adherence measures (missing any dose in the last 3 days; time since last missed dose; reasons for missed dose).<br>• Social support for taking ART<br>• Side effects<br>• ART attitudes	• Adherence improved between baseline and follow-up (missing any dose in the past 3 days decreased from 39% to 18%; time since last missed dose increased)<br>• There were no significant changes in side effects or ART attitudes.<br>• The proportion of people who anticipated the potential for problems from receiving general health messages being overheard or answered by others increased from 20% at baseline to 48% at follow-up.	25%
Uhrig (2012) [33] **Tailored SMS**	USA	To demonstrate that a tailored SMS-based intervention delivered via personal mobile phones for HIV-positive MSM aged 25 years and older, has potential to influence behavioural outcomes related to managing HIV.	Quantitative and qualitative Pre-post	SMS	HIV positive MSM (n=52)	• HIV knowledge, attitudes, and beliefs<br>• sexual behaviour<br>• social support<br>• patient involvement<br>• feasibility<br>• satisfaction	• Participants reported strong receptivity to the messages and the intervention.<br>• HIV knowledge and social support increased from baseline to follow-up.	25%

Suppl. Table 1 contd.....

Reference/ Intervention	Country	Aim	Methods/ Design	mHealth element	Population (sample size)	Outcomes and measures	Findings	Quality assessment (MMAT)
							• Among participants who received sexual risk reduction messages, there was a significant reduction in reported risk behaviours from baseline to follow-up.	
van Borek (2013) [40]<br>**WelTel Oaktree**	Canada	To determine the feasibility and acceptability of an mHEALTH intervention for improving care for HIV positive patients on highly active antiretroviral therapy (HAART) in British Columbia, Canada.	Qualitative Cross-sectional	Interactive SMS	HIV positive adults on ART (n=20)	• Thematic analysis of baseline and exit semi-structured interviews	• Intervention perceived to be feasible, acceptable, and an improvement in care. All would recommend to a peer.<br>• Benefits: someone caring, not being alone, appointment/ test reminders, help with medication and help with crises.<br>• Challenges: language, difficulties texting responses, and service provider issues/reception.<br>• Recommendations: varying frequency / content of text messages, sending messages in preferred language, appointments /medication reminders, and receiving results via text message.	NA (conference abstract)
Van der Kop (2012) [38]<br>**WelTel Kenya**	Kenya	To describe problems participants identified through mobile phone support and reasons why participants did not respond to the messages; to investigate factors associated with indicating a problem and not responding; and 3) to examine participant perceptions of the intervention.	Quantitative Secondary analysis of RCT (WelTel Kenya1 trial)	SMS	HIV positive adults (n=271)	• Factors associated with non-response<br>• Perceived barriers and benefits	• Approximately half of the participants who received the intervention responded with a problem at least once during the trial.<br>• Health issues were the primary reason for problem responses (72%).<br>• Rural residence and age were associated with indicating a problem.	75%

Suppl. Table 1 contd.....

Reference/ Intervention	Country	Aim	Methods/ Design	mHealth element	Population (sample size)	Outcomes and measures	Findings	Quality assessment (MMAT)
							• Higher educational level was associated with a decreased rate of non-response<br>• Of participants interviewed, 62% (n = 129) stated there were no barriers to the intervention<br>• Cell phone issues were the most common barrier.<br>• Benefits included reminding patients to take medication and promoting a feeling that "someone cares".	
Vidrine (2012) [53] **CPI**	USA	To describe 3-month smoking-related outcomes from an RCT of a cell phone smoking intervention.	Quantitative RCT	Phone call	HIV positive adults, smokers, willing to set a quit date within 7 days (n=474)	• Abstinence (7-day 24-hr, 30-day, continuous and mean length of longest period of abstinence)	• At follow-up, participants in the CPI group were significantly more likely to be abstinent compared with those in the UC group.<br>• Mean length of longest period of abstinence during the 3-month follow-up was 14.7 days in the CPI group versus 6.6 days in the UC group<br>• The proportion of participants who made a quit attempt was not significantly different in the two groups.	50%
Vidrine (2015) [54] **CPI**	USA	To evaluate mediators of a cell phone-delivered intervention for HIV-positive smoker	Quantitative RCT	Phone call	HIV positive adults, smokers, willing to set a quit date within 7 days (n=350)	• Abstinence at 3-months (self-reported abstinence within the past 7 days and a CO level <7 ppm).<br>• depressive symptoms (Centres for Epidemiologic Studies Depression (CES-D) scale)	• Abstinence rates in the UC and CPI groups were 4.7% and 15.7% respectively.<br>• The CPI group experienced a decline in depression and anxiety.<br>• Self-efficacy increased for the CPI group and declined for the UC group.	25%

Suppl. Table 1 contd.....

**Reference/ Intervention**	**Country**	**Aim**	**Methods/ Design**	**mHealth element**	**Population (sample size)**	**Outcomes and measures**	**Findings**	**Quality assessment (MMAT)**
						• anxiety (state component of the State-Trait Anxiety Inventory)<br>• social support (Interpersonal Support Evaluation List)<br>• quit motivation (Reasons for Quitting Questionnaire<br>• self-efficacy (9-item scale)	• Only self-efficacy met the predefined criteria for mediation.<br>• The effect of the cell phone intervention on smoking abstinence through change in self-efficacy was statistically significant and accounted for 17% of the total effect of the intervention on abstinence.	
Walsh (2012) [41]<br>**TRX Care system**	UK	To determine the acceptability of an SMS adherence support system in a pilot study.	Quantitative Single arm cross over study	SMS	HIV positive adults, on ART (n=14)	• 24 week adherence<br>• Satisfaction with the TRXCare system	• At baseline adherence was 99.5% and remained high at 98% at week 24.<br>• The median number of reminders per patient was 14 (range 1-43).<br>• 64% were satisfied with the system.<br>• 50% found the text reminders & overall system useful<br>• 67% found the verbal feedback useful.<br>• 54% found the pill -box inconvenient or that it made more difficult to take HAART regularly.<br>• 55% found reminders irritating.	NA (conference poster)
Winstead-Derlega (2012) [59]<br>**Educational videos**	USA	To explore the feasibility and potential impact of providing peer health messages using mobile technology in an academic medical centre's HIV clinic serving a primarily rural southern U.S. patient population	Quantitative Pre-post	Video	HIV positive patients (n=30)	• Attitudes towards engagement in HIV care and comfort with HIV disclosure.<br>• Satisfaction with intervention.	• 70% of patients reported that the videos were somewhat to very "helpful and meaningful,"<br>• 60% participants reported the videos made clinic visits more enjoyable<br>• Half reported that the video messages made them feel stronger or made them more committed to attend clinic.	25%

Suppl. Table 1 contd.....

Reference/ Intervention	Country	Aim	Methods/ Design	mHealth element	Population (sample size)	Outcomes and measures	Findings	Quality assessment (MMAT)
							• At baseline participants reported positive attitudes towards engagement in care but scored neutrally on HIV disclosure.<br>• There were no statistically significant changes between baseline and follow-up attitudes.	

Supplementary Table 2. Published protocols.

Author (year)	Country	Aim	Design	mHealth component	Description of intervention	Population (target sample)	Outcomes and measures
L'Engle (2015) [61]	Ghana	To evaluate the impact, implementation, and cost effectiveness of a mobile phone adherence support intervention delivered to patients on ART in large-scale public sector health services in Ghana	RCT	SMS	LifeLine – a one-way text messaging service sends daily ART reminders at no cost. Messages do not mention HIV or ART.	HIV positive patients on ART for at least 6 months, own mobile phone (1600)	ART adherence (VAS and pharmacy refills), viral load (laboratory testing), retention in care (clinical care data) and condom use (clinical care data).
Christopoulos (2014) [62]	USA	To investigate whether a text messaging intervention that supports healthy behaviours, encourages consistent engagement with care, and promotes antiretroviral persistence can improve retention in care and virologic suppression	RCT	SMS	Connect4Care - text message appointment reminders vs. text message appointment reminders plus thrice-weekly supportive, informational, and motivational text messages (includes information on request)	HIV positive patients, own mobile phone, new to clinic or history of poor retention in care	Primary efficacy outcome: virologic suppression at 12 months. Secondary outcomes: retention in HIV care (kept and missed primary care visits). Depression; perceived social support; health related quality of life (RAND-36); health care empowerment. Process outcomes: text message response rate and percent of time in study without cell phone service.
Kurth (2013) [64]	USA	To evaluate the combined effect of computerized motivational interview counselling and post-release SMS text message reminders to increase antiretroviral therapy (ART) adherence and linkage and retention in care among HIV-infected persons involved in the criminal justice system	RCT	SMS	The CARE+ Corrections Intervention. A counselling session delivered on the CARE+ platform prior or soon after jail release; SMS text messaging intervention delivered in the community after release; Text messages related to appointment reminders; medication adherence, HIV secondary prevention; barriers to care. Participants choose from prescribed messages and have the opportunity to develop customized messages (to increase confidentiality/ motivation).	HIV-infected persons involved in the criminal justice system (320)	Undetectable viral load<br>Appointment attendance<br>Self-reported adherence to ART

Suppl. Table 2 contd.....

Author (year)	Country	Aim	Design	mHealth component	Description of intervention	Population (target sample)	Outcomes and measures
Van der Kop (2013) [63]	Kenya	To determine the effect of the WelTel intervention compared with standard care on 12-month retention in care in patients newly diagnosed with HIV.	RCT	SMS	WelTel Retain – weekly SMS to check how patients are doing and allow them to identify whether assistance is required. Two way messages – participants asked Mambo (how are you?) and reply OK or Sawa; or Shida if there is a problem. All patients who indicate that there is a problem will be followed up by a nurse experienced in HIV care. Those who do not respond within 48h are telephoned by a nurse experienced in HIV care.	HIV positive adults not previously assessed for ART eligibility	Retention in care measured by follow-up appointment attendance. Secondary outcomes – ART initiation (if eligible); time to ART initiation; 6-month retention in care; mean proportion of scheduled appointments kept; level of engagement (non-engagers; sporadic users; regular users measured by appointment attendance); social support (Medical Outcomes Study Social Support Survey); satisfaction with care; adverse events (number and grade); all-cause mortality.

Supplementary Table 3. Currently funded studies (ClinicalTrials.gov).

Responsible person/ Institution	Study period	Method	Summary of the intervention	Target population	mHealth component	Outcome measures
Frimpong; Columbia University, USA	2015-2016	Single Arm Intervention Study	Mobile phone-based health (mHealth) text messaging intervention to improve engagement in HIV primary care among substance abusing populations with HIV.	Clients of methadone maintenance treatment programs who are living with HIV.	SMS	Acceptability of the intervention (Technology Assistance Model (TAM) scale)<br>Feasibility of the intervention (recruitment and attrition rates)<br>Self-efficacy to manage HIV infection (HIV-ASES)<br>HIV appointment adherence questionnaire<br>Improvements in HIV knowledge (HIV Knowledge Questionnaire (HIV-KQ-18)
Holstad; Emory University, USA	2013-2016	RCT	Live Network mobile phone app consists of a pre-recorded talk show where a DJ invites questions and comments from callers on HIV medication, adherence and self-management. These questions are posed to HIV experts, and songs are played which reinforce and augment the issues raised. The Live Network will be compared with an app consisting of pre-recorded songs providing information on various topics not related to health.	HIV positive adults living in rural areas	App	Adherence to ART; ARV drug levels in hair sample analysis; symptoms, symptom management; mediators: self-efficacy; outcome expectancies; personal goal setting. Moderators: depression; health literacy. CD4 count; viral load suppression
Shriver; University of Connecticut Health Center, USA	2012-2017	RCT	Three 16-week treatment conditions: (1) standard care; (2) standard care + cell phone-based adherence reminders; or (3) standard care + SMS adherence reminders with contingency management (monetary reinforcement for sending time/date stamped self-videos showing ingestion of ART)	HIV positive adults initiating a new ART regimen	SMS	Self-report of medication adherence

Suppl. Table 3 contd.....

Responsible person/ Institution	Study period	Method	Summary of the intervention	Target population	mHealth component	Outcome measures
Brown; Rhode Island Hotel, USA	2012-2016	RCT	A Pilot Gaming Adherence Program for Youth Living With HIV Behavioural: IMB Gaming Adherence Intervention Combination of smart pill bottle cap with mobile gaming application tailored for those living with HIV Behavioural: Enhanced treatment as usual Smart pill bottle cap and mobile phone but no game	HIV positive young people (age 14-26), receiving ART	SMS/ App	Number of times the smart pill bottle was opened; number of kept medical appointments; HIV-1 viral load; IMB ART Behavioural Skills Scale; Self- efficacy for adherence to medical care related to antiretroviral medication and treatment
RAND, The AIDS Support Organisation; Uganda	2015-2016	RCT	SMS as an Incentive To Adhere (SITA) . Intervention 1- weekly SMS feedback on participants' own adherence that week compared to receiving feedback on participants own adherence and adherence exhibited by other participants	HIV positive young people (age 15-24); taking ART	SMS	Self-reported adherence

Supplementary Table 4. Characteristics of published articles (n=41).

Category	Subcategory	N (%)
Article type	Peer reviewed paper	31 (76)
	Conference abstract	7 (17)
	Conference poster	2 (5)
	Letter to editors	1 (2)
Country	North America	23 (56)
	Africa	7 (17)
	Asia	6 (15)
	South America	2 (5)
	Europe	2 (5)
	New Zealand	1 (2)
Specific population	HIV positive adults	14 (34)
	Low adherence to ART	6 (15)
	Young people	5 (12)
	Smokers/ risk drinkers/ drug users	5 (12)
	Vulnerable adults	4 (10)
	Men who have sex with men	3 (7)
	ARV naïve adults	2 (5)
	Women	1 (2)
	PLWH with comorbid bipolar disorder	1 (2)
Methodology	Quantitative	32 (78)
	Qualitative	3 (7)
	Mixed quantitative and qualitative	6 (15)
Design	RCT	14 (34)
	Pre-post	13 (32)
	Cross-sectional	12 (29)
	Retrospective	2 (5)

Supplementary Table 5. Features of interventions included in published studies (n=28).

Category	Subcategory	N (%)
Focus of intervention	Medication adherence	16 (57)
	Engagement with care	3 (11)
	Smoking cessation	1 (4)
	Complex self-management	8 (29)
Delivery method	SMS only	16 (57)

Suppl. Table 5 contd.....

Category	Subcategory	N (%)
	App	5 (18)
	Phone calls	3 (11)
	IVR	1 (4)
	SMS + IVR	1 (4)
	Video	1 (4)
	Choice of delivery modes	1 (4)
Type of evaluation	Feasibility	9 (32)
	Evaluative	19 (68)
Theory-derived	Yes	5 (18)
	No/ Not stated	23 (82)
User involvement	Yes	14 (50)
	No/ Not stated	14 (50)
Incentive	Yes (*e.g.* expenses, phone/ipod or incentive to enrol/ complete the study)	14 (50)
	No/ Not stated	14 (50)
Personalised	Yes	20 (71)
	No/ Not stated	8 (29)
Covert	Yes	15 (54)
	No/ Not stated	13 (46)
Functions included	Medication reminder	16 (57)
	Behaviour monitoring	13 (46)
	Patient provider communication	10 (36)
	Personalised feedback	8 (29)
	Appointment reminder	6 (21)
	Motivational messages	4 (14)
	Lab reports	3 (11)
	Information provision	2 (7)
	Medication log	2 (7)
	Mood monitoring	1 (4)
	Physical and mental health monitoring	1 (4)
	Medication review	1 (4)
	Signposting to other resources	1 (4)
	Peer education	1 (4)
	Smoking cessation counselling	1 (4)
Number of functions	1	8 (29)
	2	6 (21)
	3	8 (29)
	4	4 (14)
	5	1 (4)
	6	1 (4)

Supplementary Table 6. Overview of the 16 SMS-based interventions.

Reference	Description of intervention	Incentive for participants	Underlying theoretical model	User involvement in intervention development	Covert?	Personalised?	Two-way?	Functions	Successful?
Da Costa (2012) [25]	**The HIV Alert System (HIVAS)**	None	Not stated	No (The content of the SMS message was chosen by consensus by the multidisciplinary team involved in patient care and the researchers)	Yes – no mention of HIV	No	No (participants asked not to respond)	• Medication reminder	Adherence improved (not powered for significance testing)

Suppl. Table 6 contd.....

**Reference**	**Description of intervention**	**Incentive for participants**	**Underlying theoretical model**	**User involvement in intervention development**	**Covert?**	**Personalised?**	**Two-way?**	**Functions**	**Successful?**
	SMS message sent automatically before the last medication dose of the day on alternate weekdays and every Saturday and Sunday for 4 months. The messages read 'The UNIFESP informs: take good care of your health'.								
Dowshen (2012) [27]	**Intelecare** Personalised text message co-designed with the patient, delivered through daily at specified times, together with a follow-up message 1 hour message, asking whether participants had taken the medication and to respond with a 1 if they had taken the medication and a 2 if they did not.	US $40 incentive at each visit to compensate for time, transportation costs, and any additional charges incurred from the daily text messages.	Not stated	Not stated (although patients choose their own message)	Patients were encouraged to consider developing messages that would respect their privacy if they did not want to disclose their HIV diagnosis.	Yes - patients worked with the study coordinator to design their own personalized SMS reminder messages	Yes – 1 if taken med, 2 if not.	• Medication reminder<br>• Behaviour monitoring	Yes – significant increase in adherence
Hailey (2013) [30]	**STAR TRACK Adherence to Medication Program (STAMP)** Stage 1: patients were sent covert SMS messages as a reminder to take daily medications (*e.g.*, How are you today?). Patients text staff to confirm they took their medication.	If participants didn't have a mobile they would have received assistance from clinical support staff to obtain one if they had a low income.	Not stated	Not stated	Yes	No	Yes – patients reply to text/ video themselves taking medication	• Medication reminder<br>• Behaviour monitoring<br>• Patient provider communication	Medication adherence reported to have increased (no significance testing)

Suppl. Table 6 contd.....

Reference	Description of intervention	Incentive for participants	Underlying theoretical model	User involvement in intervention development	Covert?	Personalised?	Two-way?	Functions	Successful?
	Stage 2 (for unresponsive patients): patients send video text messages of themselves taking medication as a form of directly observed therapy. Dedicated clinic cell phone with 24-hour staff coverage.								
La Grange (2012) [28]	**CareSpeak** Participants received an automated, tailored, SMS message using the CareSpeak mHealth adherence monitoring platform to remind them to take their medication on a daily basis. (*e.g.* “Take your meds” or “Take your multi-vitamin”). After taking their medication, youth replied to the original text via a two-way SMS feature of CareSpeak.	Not stated	Not stated	Not stated	Option for covert message *e.g.* “take your multivitamin”	Participants chose the content of their reminder text.	Yes	• Medication reminder<br>• Behaviour monitoring	Not assessed
Mbuagbaw (2013) Mbuagbaw (2012) Mbuagbaw (2012) [31, 44, 68]	**Cameroon Mobile Phone SMS (CAMPS)** Weekly SMS intervention. Motivational content, with a reminder component and a number to call for help (*e.g.* “You are important to your family. Please remember to take your medication. You can call us at this number: +237 xxxx xxxx.”)	None	Health Belief Model	Yes - messages were developed based on data collected from focus group discussions	Yes - the messages made no mention of HIV.	In either French or English, based on the participant’s language preference.	Yes - option for participants to respond to the SMS by text or phone call.	• Medication reminder<br>• Motivational messages<br>• Patient provider communication	No – no significant effect on adherence

Suppl. Table 6 contd.....

Reference	Description of intervention	Incentive for participants	Underlying theoretical model	User involvement in intervention development	Covert?	Personalised?	Two-way?	Functions	Successful?
Moore (2015) [23]	**Individualized Texting for Adherence Building (iTAB)** Automated, personalized, two-way, text messaging system designed to send text messages directly to participant's mobile phones, including medication reminders. Reminders were sent for both HIV and bipolar medications, once daily or twice daily, depending on the regimen instructions. a reminder message might read, "John, your meds r important. It is time to take ur meds. Take ur big blue pill now. Pls reply (A) took (D) didn't (G) snooze." A reinforcement message might read, "Great job! Ur current adherence: 75%. Adhr when u take ur next dose: 80% (4/5 doses)"	Participants received monetary compensation for both the initial and follow-up assessments. They were also reimbursed for costs incurred for using their own phones or if necessary provided with a mobile phone with a comprehensive texting plan for the duration of the study.	Theory of planned behaviour	Yes - Sample TPB reminder messages were provided to pilot HIV+ persons who provided feedback during the initial development and refinement phase of iTAB	Medications were described (*e.g.* big white pill) to avoid using potentially stigmatizing medication names.	The participant and investigator selected appropriate reminder messages, reinforcement messages (*e.g.* 'U R awesome! Keep up the good work!') and descriptions of the medications for use in the messages. Personalised feedback provided in text messages (*e.g.* adherence rate)	Yes – participants reply to indicate whether they took medication and reinforcement message is tailored accordingly.	• Medication reminder<br>• Behaviour monitoring<br>• Motivational messages<br>• Personalised feedback	Yes – on timing adherence for ART
Murray (2015) Murray (2015) Murray (2013) Smillie (2014) van Borek (2013) [26, 39, 40, 43, 69]	**WelTel Oaktree** Participants received weekly text messages stating "How are you?" A clinic nurse responded to non- and negative responses.	Participants were given a cell phone with unlimited texting if they did not have one.	Not stated	Yes - They published a study in 2012 looking at patients' views about an SMS intervention	HCPs never texted information relating to HIV status or the clinic unless asked explicitly to do so by the participant.	No	Yes - Participants were instructed to respond if they were "OK" or "Not OK" but open responses were accepted and the software captured all texting content.	• Patient-provider communication	Yes – significant improvement in adherence and viral load.

Suppl. Table 6 contd.....

Reference	Description of intervention	Incentive for participants	Underlying theoretical model	User involvement in intervention development	Covert?	Personalised?	Two-way?	Functions	Successful?
Reid (2014) [42]	**SMS reminders** SMS reminders when medication refills were due	None reported	Not stated	Not stated	Not stated	No	No	• Medication reminder	Not assessed
Sabin (2015) [24]	**China Adherence through Technology Study RCT** Intervention subjects received adherence counselling as clinically indicated, and an SMS mobile phone reminder sent whenever the Wisepill system failed to detect a device opening by 30 minutes past a scheduled dose time. The text messages were personalized, with subjects selecting from a list of 10 options developed jointly by clinicians and patients, including "carry on, carry on!" and "be healthy, have a happy family."When seen monthly in clinic, subjects with prior-month adherence <95% received a behaviourally targeted counselling session with a counsellor guided by a detailed day-to-day adherence performance report with a visual display of doses taken and summary of doses taken on time, off time, and missed in the previous month.	All subjects received 150 yuan (approximately US $25) monthly as reimbursement for lost work time and travel costs associated with study participation.	Not stated	Yes - Patients were involved in developing the text options.	To prevent disclosure of HIV, text reminders did not refer to HIV, ART, or other disease-related topics.	Patients chose one of 10 messages.	No	• Medication reminders • Motivational messages • Behaviour monitoring • Personalised feedback	Yes – significant impact on adherence

Suppl. Table 6 contd.....

Reference	Description of intervention	Incentive for participants	Underlying theoretical model	User involvement in intervention development	Covert?	Personalised?	Two-way?	Functions	Successful?
Van der Kop (2012) [38]	**WelTel Kenya** For 12 months, every Monday morning, a clinician (nurse) sent the text message "Mambo?" ("How are you?") to patients in the intervention group to inquire about their status. Patients were instructed to respond within two days either that they were doing well ("Sawa.") or had a problem ("Shida."). The clinician called and provided triage to patients who indicated a problem or failed to respond.	None reported	Not stated	Not stated	No mention of HIV	No	Yes - Patients were instructed to respond within two days either that they were doing well ("Sawa.") or had a problem ("Shida.")	• Patient-provider communication	Not assessed
Walsh (2012) [41]	**TRX Care system** The SMART dispenser is an intelligent reminder system provided by TRX Care. A slim-line medication organiser sends a mobile phone signal to a remote server when opened. It monitors dose events and texts reminders for missed doses & positive feedback on adherence. From weeks 13-24 only, text reminders were sent to the subject's own mobile 'phone if a dose was late; weekly text messages were sent to reinforce adherent behaviour.	None reported	Not stated	Not stated	Not stated	Yes - In response to individual's pattern of medication taking. The definitions of a late dose were set in collaboration with the patient.	No	• Medication reminder<br>• Behaviour monitoring<br>• Personalised feedback	Not assessed

Suppl. Table 6 contd.....

Reference	Description of intervention	Incentive for participants	Underlying theoretical model	User involvement in intervention development	Covert?	Personalised?	Two-way?	Functions	Successful?
	At weeks 12 & 24 subjects received verbal feedback on their adherence from the study team using data held on the system's server (including graphs of dosing patterns).								
Norton (2014) [35]	**Call-em-All** Participants had to opt-in to the online texting service (Call-em-All) by sending a text message from their cell phones. Text messages were sent 1 day prior to the appointment date and read “Remember: you have a doctor’s appointment tomorrow.”	None reported	Not stated	Not stated	Yes - HIV not mentioned	No	No	• Appointment reminder	No – no significant difference in attendance
Farmer (2014) [37]	**SMS appointment reminders** Text reminders for patients with pre-booked appointments sent 2 days before the appointment. Message states: ‘You have an appointment on <ApptDate> <ApptTime>. If it is your first appointment please arrive early. Ring (clinic telephone number) to cancel/change. Do not text back. From CMH’.	None	None stated	Not stated	Yes - HIV/ sexual health not mentioned.	Only with appointment details.	No	• Appointment reminder	No – no change in HIV attendance rates

Suppl. Table 6 contd.....

Reference	Description of intervention	Incentive for participants	Underlying theoretical model	User involvement in intervention development	Covert?	Personalised?	Two-way?	Functions	Successful?
	Texts are sent free of charge through nhs.net which provides a free texting service to all NHS service providers. Automated texting is an integrated function of the patient management system.								
Siedner (2014) Siedner (2015) [36, 70]	**SMS intervention** Participants with a normal CD4 laboratory result received a single message indicating a normal result and requesting return on the date of their next visit. Those with abnormal results received one of three SMS text message formats:<br>1. Direct message: "This is an important message from your doctor. You had an abnormal test result. You should return to clinic as soon as possible"<br>2.An identical message as the direct message, but prompted by an initial requirement to enter a 4-digit personal identification number.<br>3. A coded message which was explained on enrolment to indicate an abnormal test result and signify early return to clinic:	They provided transportation reimbursement (~$6USD) for those who needed to return to the clinic following an abnormal result.	None stated	Yes - Based on a conceptual framework derived from a preliminary survey of clinic patients, conducted to understand barriers to linkage and acceptability of SMS text message-based health communications	Varied across conditions	The number and timing of messages was determined by their scheduling preferences on enrolment.	No	• Patient-provider communication	Yes- significant impact on time to clinic return and ART uptake

Suppl. Table 6 contd.....

Reference	Description of intervention	Incentive for participants	Underlying theoretical model	User involvement in intervention development	Covert?	Personalised?	Two-way?	Functions	Successful?
	"ABCDEFG" Participants with abnormal test results were eligible to receive messages daily for up to a maximum of 7 days.								
Uhrig (2012) Lewis (2013) Furberg (2012) [32 - 34]	**Tailored SMS** Tailored SMS-based intervention focused on changing multiple behavioural outcomes among people living with HIV using bidirectional messaging and tailoring of content throughout the intervention (i.e., dynamic tailoring). Participants responded to questions via SMS throughout their exposure to the intervention.	Each participant received an incentive of $25 upon enrolment and $10 per month for the 3-month study period.	Not stated	Yes - A set of draft messages were previously developed using a systematic formative research process. Messages were qualitatively pretested with eight members of the target population. Feedback was incorporated into the final messages.	Not reported	A mix of tailored and non-tailored messages. E.g those reporting non-adherence or starting a new regimen received reminder messages tailored to their individual ART regimen. Participants who were adherent received weekly adherence messages encouraging them to keep taking medications as prescribed.	Yes – participants provided information used to tailor messages (i.e., dynamic tailoring).	• Medication reminder<br>• Motivational messages<br>• Behaviour monitoring<br>• Appointment reminders<br>• Information provision (sexual health, substance use, exercise, nutrition, mental health, social support, patient involvement in care)<br>• Signposting to other resources	Yes – improved adherence, viral load, CD4, social support, risk reduction, HIV knowledge
Ingersoll (2014) [29]	**Treatment Extension by Text (TxText)** A daily two-way automated text messaging service that assesses ART adherence, substance use, and mood, and delivers self-created personalized intervention messages. Staff can also send messages (*e.g.* appointment reminders).	Participants were given free study phones and discounted monthly service plans for the study)	Not stated	Yes – surveys, individual and small group interviews and user testing/feedback contributed to development.	Mood and medication use were not covert, drug use was assessed using the coded question "How were the skies in the past 24 hours?" Participants respond SKIES clear if they are reporting no drinking or drug use in the past 24 hours, SKIES cloudy if they smoked marijuana, rainy if they drank alcohol,	Participants design their intervention messages for each targeted issue (nonadherence, poor mood, substance use)	Yes – participants respond to questions on mood, drug use and medication adherence.	• Behaviour monitoring<br>• Mood monitoring<br>• Medication reminders<br>• Appointment reminders<br>• Personalised feedback	Not assessed

Suppl. Table 6 contd.....

Reference	Description of intervention	Incentive for participants	Underlying theoretical model	User involvement in intervention development	Covert?	Personalised?	Two-way?	Functions	Successful?
					snowy if they used crack or cocaine, and other if they used other illicit drugs.				

Supplementary Table 7. Overview of the 5 app-based interventions.

Reference	Description of intervention	Incentive for participants	Underlying theoretical model	User involvement in intervention development	Covert?	Personalised?	Two-way?	Functions	Successful?
Hasin (2014) [49]	**HealthCall-S** Following a motivational interviewing session, patients used the smart phone adaptation of the Health-Call intervention to record their daily alcohol intake, medication adherence and risk taking behaviour (self-monitoring component) with one-touch link to counsellor's phone if necessary. The recorded number of drinks per day were used to generate graphs – discussed with patients in a meeting with the counsellor at 30 and 60 days (personalized feedback), and displayed on the smart phone (optional)	Patient received gift certificates as incentives (*e.g.* $25 at baseline, 30 days and 90 days, $100 at completion).	Not stated	Yes - Patient and HIV health educator-counsellor input was obtained throughout Health Call adaption process to create a more engaging, user-friendly patient procedure.	Not stated. 38% patients had some concerns about privacy.	Yes – personalized graph feedback	Yes – patient enters information and can call counsellor.	• Behaviour monitoring<br>• Personalised feedback<br>• Patient-provider communication	Yes – as good as successful IVR intervention at reducing drinking
Luque (2013) [47]	**MyMedical** MyMedical application which allows users to enter, store, and retrieve medical information	Participants received an iPod Touch at the end of the training in order to use their PHR applications.	Not stated	Yes - The study was conducted using a community-based participatory research approach (CBPR).	Password protected	Patients enter their own data (*e.g.* test results, appointment details)	Yes – patients set own reminders/ entered their own data.	• Medication log<br>• Lab reports<br>• Medication reminders<br>• Appointment reminders	Yes – improved self-efficacy for treatment adherence

Suppl. Table 7 contd.....

Reference	Description of intervention	Incentive for participants	Underlying theoretical model	User involvement in intervention development	Covert?	Personalised?	Two-way?	Functions	Successful?
	(*e.g.* medications, tests and procedures, documents). The application/ device also offered options for appointment and medication reminders								
Perera (2014) [45]	**Smartphone application incorporating personalized health-related visual imagery** Control group received a smartphone application consisting of a 24-h medication clock displaying the participant's daily ART dosing schedule, allowing participants to record when they had taken their medications. Participants in the intervention group received an augmented version of the application which, in addition to the 24-h medication clock, contained: (1) graphical representations of the estimated plasma concentrations of each of their antiretroviral medications, and (2) a personalized disease state simulation of immune activity that comprised a moving pictorial representation of the patient's CD4 count and HIV viral load (based on their most recent blood tests).	None reported	Common Sense Model of self-regulation	Not stated	Not stated	Yes – graphical representations tailored to blood test results/ adherence levels.	Yes – graphical feedback based on adherence record	• Medication log<br>• Behaviour monitoring<br>• Personalised feedback<br>• Lab reports (graphical)	Yes – significantly better adherence and viral load in intervention group

Suppl. Table 7 contd.....

Reference	Description of intervention	Incentive for participants	Underlying theoretical model	User involvement in intervention development	Covert?	Personalised?	Two-way?	Functions	Successful?
Rosen (2015) [46]	The app provides reactive, visual representations of adherence rates, viral load and CD4 counts.	Not stated	Not stated	Yes - Focus group to inform the development of the app.	Not stated	Yes – app is reactive based on adherence, viral load and CD4 count	Yes	• Lab reports<br>• Behaviour monitoring<br>• Personalised feedback	Not assessed
Swendeman (2015) [48]	**Smartphone self-monitoring** Participants complete mobile phone surveys once daily on alcohol, tobacco, and other drug (ATOD) use, sexual behaviours, and medication adherence; 4 times per day on physical and mental health-related quality of life to capture and reflect high variability in symptom experiences throughout a day; and to self-initiate stressful event reports or a text diary entry at any time. Participants complete phone surveys at times they programmed the application alarm to trigger (ie, time based reporting) as well as at any time when relevant experiences occurred (event-based reporting). A Web-based visualization portal was also available to mobile phone group participants,	Participants received a study-assigned mobile phone (a first-generation Android G1 smartphone, valued at $50). Participants were reimbursed for completing surveys (up to $170).	Not stated	Yes - Formative focus groups with PLWH (n = 29) recruited from the primary study site informed the design	No	Participants enter their own data. They can then see their responses over time.	Yes – participants enter their own data and receive personalised feedback.	• Behaviour monitoring<br>• Physical and mental health monitoring<br>• Personalised feedback	Not assessed

Suppl. Table 7 contd.....

Reference	Description of intervention	Incentive for participants	Underlying theoretical model	User involvement in intervention development	Covert?	Personalised?	Two-way?	Functions	Successful?
	which was capable of displaying their personal phone survey responses over time, by location (using a Google map of phone survey geolocation stamps), and association between variables.								

© 2017 Cooper *et al*.

This is an open access article distributed under the terms of the Creative Commons Attribution 4.0 International Public License (CC-BY 4.0), a copy of which is available at: https://creativecommons.org/licenses/by/4.0/legalcode. This license permits unrestricted use, distribution, and reproduction in any medium, provided the original author and source are credited.
